# Establishment and Assessment of New Formulas for Energy Consumption Estimation in Adult Burn Patients

**DOI:** 10.1371/journal.pone.0110409

**Published:** 2014-10-16

**Authors:** Peng Xi, Wang Kaifa, Zhang Yong, Yan Hong, Wang Chao, Song Lijuan, Wang Hongyu, Wu Dan, Jiang Hua, Wang Shiliang

**Affiliations:** 1 State Key Laboratory of Trauma, Burns and Combined Injury, Institute of Burns of PLA, Southwest Hospital, Third Military Medical University, Chongqing, P.R. China; 2 Department of Mathematics, School of Biomedical Engineering, Third Military Medical University, Chongqing, P.R. China; 3 Department of Computational Mathematics and Biostatistics, Metabolomics and Multidisciplinary Laboratory for Trauma Research, Sichuan Provincial People's Hospital, Sichuan Academy of Medical Sciences, Chengdu, China; Rutgers University, United States of America

## Abstract

**Objective:**

An accurate knowledge of energy consumption in burn patients is a prerequisite for rational nutrition therapy. This study sought to create a formula that accounts for the metabolic characteristics of adult burn patients to accurately estimate energy consumption of patients with different areas and extents of burn and at different times after injury.

**Methods:**

Resting energy expenditure (REE) data on 66 burn patients, with total body surface area (TBSA) of burns ranging from 4% to 96%, were evaluated at different times after injury. REE values were determined in patients using indirect calorimetry at days 1, 2, 3, 7, 14, 21, and 28 after injury. We then constructed a mathematical model of REE changes post-burn. Next, established two new formulas (one non-linear and the other linear) for energy consumption estimation using model-based analytical solution and regression analysis. The new formulas were compared with measured REE and commonly used formulas including those of Carlson, Xie, Curreri, and Milner to determine accuracy and reliability.

**Results:**

Comparative analysis showed that the new formulas offered significantly higher accuracy and reliability than the Milner formula, which is considered the most accurate of commonly used burn energy consumption estimate formulas. The accuracy of the new nonlinear formula (94.29%) and that of the linear formula (91.43%) were significantly higher than that of Milner formula (72.86%) when compared to measured REE (χ2  =  11.706, P  =  0.001; χ2  =  8.230, P  =  0.004, respectively). The reliabilities of the new estimation formulas were both 100% and that of Milner formula was 74.24% (χ2  =  19.513, P  =  0.000).

**Conclusion:**

The new formulas constructed in this study provide reliable simulation of the impact of the degree of burn and post-burn days on energy consumption and offer notably higher accuracy and reliability than other formulas. These formulas will help determine nutritional needs of burn patients.

**Trial Registration:**

The study was registered on Chinese Clinical Trial Registry as ChiCTR-TRC-13003806.

## Introduction

Severely burned patients have active catabolism and high levels of energy consumption that can result in progressive weight loss, immune dysfunction, visceral organ dysfunction, delayed wound healing, or even death[1]–[3]. Rational nutrition support is important for ameliorating nutritional status, reducing complications, and improving the prognosis of patients[4]–[6]. An accurate knowledge of energy consumption is necessary for development of appropriate targeted nutritional intervention[7]–[9]. Indirect calorimetry and energy consumption estimation formulas are currently used to estimate energy consumption in burn patients. Indirect calorimetry provides an accurate determination of the energy consumption of the patients; however, metabolic cars are expensive and the utilization rate is only about 66% even in developed countries like the United States[5]. In addition, frequent metabolic measurements disturb patients, increase clinical workload, and compliance is poor for patients with head and facial burns. A variety of formulas for energy consumption estimation have been developed to address these limitations. In 2002, Dickerson reviewed 46 energy estimation formulas published over the past half century and compared the formulas with actual energy consumptions in burn patients. The researchers concluded that the most accurate formulas were Milner, Zawacki, and Xie formulas[7]. In 2013, Shields carried out an in-depth analysis of the Dickerson study and compared nine commonly used energy estimation formulas; these authors argued that the Milner and Carlson formulas fit best with actual energy consumptions[8].

In 1993, our research group established a formula for energy consumption estimation based on the body surface area and the percent burned area[10], [11]. This is referred to as the Xie formula by Dickerson but in China is known as the Third Military Medical University Adult Burn Patients Energy Consumption Estimation Formula or the TMMU Formula. This formula was included in the 2005 edition of Chinese Burn Treatment Guidelines and has been adopted by many Chinese burn units for prediction of energy consumption of burn patients[12], [13]. Dickerson gave a high evaluation of the Xie formula and noted that it was particularly attractive for clinical practice[7]. After over two decades of use, we have found that although the Xie formula is simple and practical, it overestimates energy consumptions in patients with extensive burns. Our calculations showed that estimated values produced from the Xie formula are about 15%, 23%, and 40% higher than measured resting energy expenditure (REE) values in patients with total body surface areas (TBSA) burned of 31–50%, 51–70%, and 71–100%, respectively[6], [11], [12]. Another limitation is that the formula does not consider the burn course, even though energy consumption is closely correlated with burn course, especially within the first month post-burn[6], [14]–[17].

The energy consumption of burn patients is regulated by numerous factors including patient characteristics (age, sex, body surface area, nutritional status, and disease factors) and by the burn area, burn depth, and post-burn days[18], [19]. Studies have shown that there is no simple linear relationship between energy consumption and burn area and post-burn days, and the use of a simple linear equation for energy consumption estimation produces estimates that deviate greatly from actual values[14], [16], [20]. However, all the energy estimation formulas currently available are based on linear equations, and most of the formulas (except for Miler) do not account for variation due to the number of post-burn days[7]–[9].

In this research, indirect calorimetry was used to determine energy consumption data from patients with different areas of burn and at different times after burn, and we sought to build a mathematical model that reflected changes in the rate of energy consumption over time. The non-linear formula developed for energy consumption estimation accurately reflects different burn areas and number of days since injury. In order to facilitate clinical use, we performed piecewise linear fitting for the formula and generated four simplified linear estimation formulas that are applicable to patients with different burn areas and at different times post injury. The practical and accurate formulas for energy consumption estimation reported here fit well with the metabolic characteristics of the Chinese people and will enable rational nutritional support of burn patients.

## Materials and Methods

### Ethics statement

The protocol of the study was approved by the Committee of Medical Ethics of the Southwest Hospital of The Third Military Medical University (approval number: KY201312). All patients or legal representatives were informed of the aims and methods of study and signed a written informed consent before the start of the experiment. The study was registered on Chinese Clinical Trial Registry (registration number: ChiCTR-TRC -13003806).

### Subjects

Sixty-six burn patients (48 men and 18 women) were enrolled in the study; ages ranged from 18 to 52 years, and TBSA affected ranged from 4% to 96%. Patient characteristics are summarized in Table 1. The patients were divided into ten groups depending on TBSA at 10% intervals with each group containing 4 to 9 patients. Patients burned with chemicals or with electrical burns, patients with inhalation injuries, or patients with severe diseases of heart, liver, kidney, or hematopoietic systems or with metabolic diseases such as diabetes or hyperthyroidism before injury were excluded. All patients were admitted within 24 hours of injury and were immediately given anti-shock fluid resuscitation. For patients with TBSA> 30%, the wounds were treated with silver sulfadiazine for eschar preservation, and antibiotics were given systemically. Eschar excision and skin grafting were begun three days after burn injury and were performed 3 to 4 times within the next month to gradually close the wounds. For patients with minor burns (TBSA <30%), the wounds were treated with Iodophor after debridement and were semi-exposed for eschar preservation.

**Table 1:** Subject characteristics (n = 66, M = 48, F = 18).

Characteristic	Mean	Standard deviation	Range
Age (yr)	31.06	8.79	18∼52
Height (m)	1.63	0.05	1.50∼1.71
Weight (kg)	55.04	5.15	45∼70
BSA (m^2^)	1.58	0.09	1.37∼1.79
TBSA (%)	45.65	28.17	4∼96
Third degree (%)	24.00	21.91	1∼80

Resting energy expenditure (RRE) was determined at 1, 2, 3, 7, 14, 21, and 28 days post-burn using indirect calorimetry. Briefly, the room temperature was kept at 28–30°C. Before determination, patients were allowed to rest at least 20 min in a supine position. Patients wore the mask for 10 min for adaptation prior to measurement. REE values were calculated through analysis of consumed oxygen and exhaled carbon dioxide. Patients did not receive surgery in the two days before REE determination. REE was measured at 9–10 a.m. and again at 5–6 p.m. Each evaluation lasted no less than 30 minutes. The average value of the two tests was used as the REE value of the day. These data are summarized in Table 2, and the detailed information is put in File S1.

**Table 2:** Resting energy expenditures of burn patients grouped based on extent TBSA at indicated post-burn day (PBD).

TBSA(%)	REE(kcal·m^−2^·day^−1^)						
	PBD 1	PBD 2	PBD 3	PBD 7	PBD 14	PBD 21	PBD 28
1–10	1179±107	1197±130	1248±103	1263±92	1216±56	1118±84	1074±49
11–20	1229±90	1259±86	1317±90	1333±85	1363±78	1245±100	1135±88
21–30	1268±140	1296±142	1352±153	1412±140	1428±138	1276±60	1220±73
31–40	1290±111	1326±97	1447±97	1495±93	1522±93	1491±95	1268±77
41–50	1325±95	1408±80	1504±67	1560±68	1676±35	1552±72	1376±71
51–60	1296±76	1402±59	1488±57	1587±88	1704±91	1688±54	1573±73
61–70	1224±88	1296±76	1555±86	1676±79	1728±82	1764±81	1640±94
71–80	1086±99	1218±91	1368±96	1620±82	1710±60	1872±52	1824±52
81–90	1025±36	1268±56	1364±51	1638±74	1728±71	1914±82	1968±71
91–100	996±46	1128±34	1326±41	1602±60	1746±49	1830±60	1902±41

### Establishment of a nonlinear estimation formula

**Figure 1:**
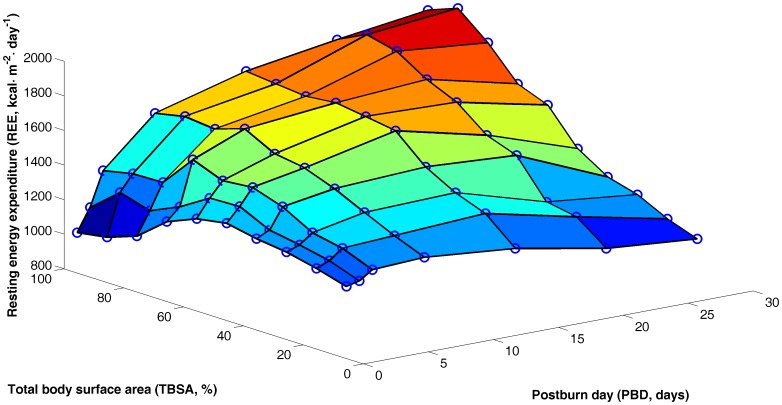
Three-dimensional display of the average REE data of burned patients plotted vs. TBSA and PBD. Circles represent average REE measurements.



The plot of REE per unit body surface area (BSA, m^2^) in burn patients as a function of TBSA and post-burn day (PBD) showed several S-shaped curves (Figure 1). Accordingly, we assumed that the rate of change of REE as a function of TBSA and PBD satisfies the following differential equation:

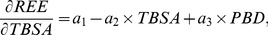
(1)

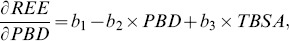
(2)


The definitions of the parameters and the units are given in Table 3.

**Table 3:** Definitions and units of the parameters in equations (1) and (2).

Parameter	Definition	Unit
*a_1_*	intrinsic rate of increase of REE as a function of TBSA	kcal·m^−2^·TBSA^−1^
*a_2_*	metabolic inhibition coefficient of TBSA for the rate of change of REE after exceeding the metabolism limit	kcal·m^−2^·TBSA^−2^
*a_3_*	synergistic coefficient of PBD for REE as a function of TBSA	kcal·m^−2^·TBSA^−1^·PBD^−1^
*b_1_*	intrinsic rate of increase in REE as a function of PBD	kcal·m^−2^·PBD^−1^
*b_2_*	metabolic inhibition coefficient of PBD for the rate of change of REE after exceeding the metabolism limit	kcal·m^−2^·PBD^−2^
*b_3_*	synergistic coefficient of TBSA for REE as a function of PBD	kcal·m^−2^·TBSA^−1^·PBD^−1^

Since equations (1) and (2) are linear differential equations, the analytical solution is:
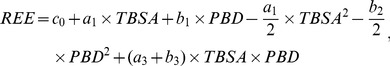
(3)wherein 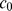 indicates daily REE level required by healthy humans (kcal • m^−2^). Formula (3) is a non-linear theoretical formula that uses both TBSA and PBD to predict REE per unit body surface area (m^2^).

### Establishment and simplification of linear estimation formulas

Given the measured REE changes after injury in the subjects evaluated, we categorized patients as those with TBSA> 70% and those with TBSA ≤ 70%. We further distinguished between two time periods: 0–14 days post-burn and 15–28 days post-burn. Based on these divisions, we built linear estimation formulas appropriate for these types of patients using multiple regression analysis. In order to make the formula simple and practical, each of the estimation parameters in the above formula was rounded where appropriate.

### Comparison of estimation formulas

The newly built formula for energy consumption estimation was compared with commonly used formulas including those of Carlson, Xie, Curreri, and Milner (Table 4) for accuracy and reliability by determining to what extent the estimates derived from these formulas deviated from actual REE measurements. Based on clinical practice, 80% to 120% of the actual energy consumption was adopted as the acceptable accurate range. The extent to which the estimates from these formulas matched the actual energy consumptions of burn patients was assessed by calculating whether values estimated with each of the formulas fell within the acceptable range.

**Table 4:** Formulas commonly used in clinical practice.

Formula	Expression
Carlson[8]	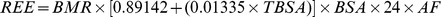
Xie[7], [10]	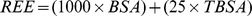
Curreri[5], [7]	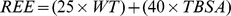
Milner[8], [21]	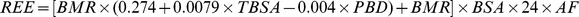

**Notes:** BMR, basal metabolic rate in healthy subjects; BSA, body surface area; AF, activity factor (typically 1.2–1.4). BMR (in kcal/m^2^/hr) was determined using the Fleisch equation (healthy population, 1951):

Men: 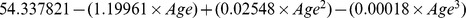

Women: 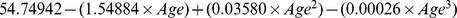

BSA (in m^2^) is the square root of (HT×WT)/3600. Here HT is height in cm, and WT is weight in kg.

As Carlson, Xie, and Curreri formulas do not consider the PBD variable, we averaged the actual REE measurements at seven different PBD points and compared the average with estimated values from the formulas derived in this study and from the Milner formula. The non-linear and linear formulas developed here and the Milner formula include the time variable, therefore we considered the impact of both TBSA and PBD simultaneously. A total of 70 different combinations of TBSA and PBD were available (ten TBSA intervals multiplied by seven PBD points). Finally, the overall reliability of the estimation formulas was evaluated by determining the extent to which the estimates matched the actual REE measurements in the 66 patients in our study group.

### Statistical analysis

All data are presented as means ± standard deviation, and all data passed a normal distribution test. Using the data in Table 2 and regression analysis, we obtained the values of the parameters in the new non-linear and linear formulas for energy consumption estimation. Using the coefficient of determination and F-statistic, we determined the goodness of fit of the regression equations with the measured values. The accuracy of formulas and their overall reliability were compared using the chi-square test, and Fisher's exact test was performed for correction of continuity when the total sample size was less than 40 or the theoretical frequency was less than 5. Statistical analysis was performed with SPSS (version 17.0), and a two-tailed probability value of less than 0.05 was considered statistically significant.

## Results

### Nonlinear estimation formula

Using data from Table 2 and the formula (3) and using multiple linear regression, we estimated values of the parameters TBSA, PBD, and BSA. The values of the coefficient of determination 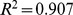 and 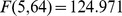 (*P*  =  0.000) indicated that the formula fit well with the measured data. Formula (3) was designed to estimate the REE per unit BSA, which was multiplied by body surface area BSA, to generate the nonlinear estimation formula that predicts REE of burn patients using PBD and TBSA:
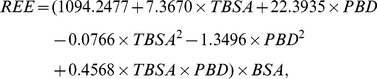
(4)


### Linear estimation formulas based on TBSA and PBD

Using multiple linear regression, we also obtained linear formulas for energy consumption estimation based on TBSA and on PBD (Table 5). For convenience in clinical applications, the coefficients in the linear formulas were rounded to generate the following simplified linear formula for energy consumption estimation:
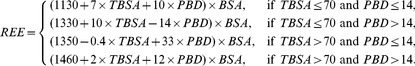
(5)


**Table 5:** Results of the multiple linear regression for energy consumption estimation.

TBSA (%)	PBD (days)	
	PBD≤14	PBD>14
≤70%	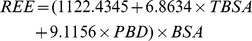 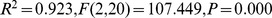	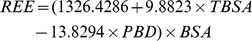 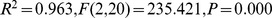
>70%	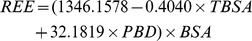 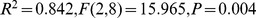	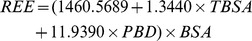 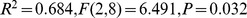

**Figure 2:**
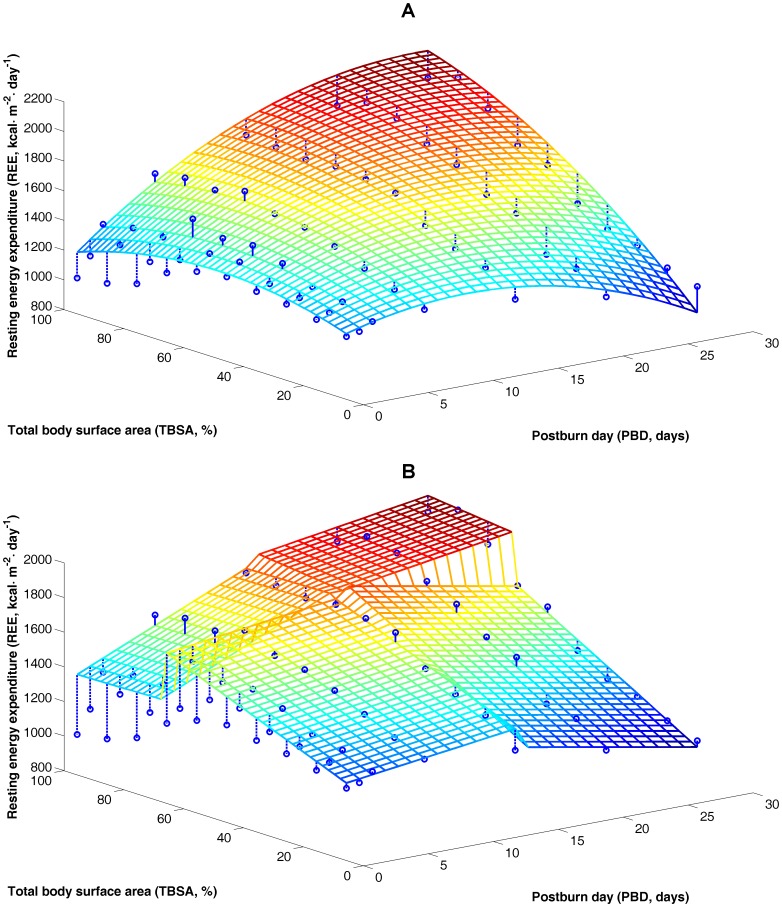
Comparison of estimates from energy consumption estimation formulas and experimental data. (A) Non-linear estimation formula (4) and (B) linear estimation formula (5). The circles represent average REE measurements for each patient.

We plotted the solution surfaces of the non-linear estimation formula (4) and the simplified linear estimation formula (5) using Matlab 7.0. As shown in Figure 2, we found that the solution surfaces of our new formulas fit well to the experimental data (Table 2), indicating that these formulas offer reliable simulation of REE changes in patients with different burn areas and at different times after injury.

### Application and comparison of formulas

**Figure 3:**
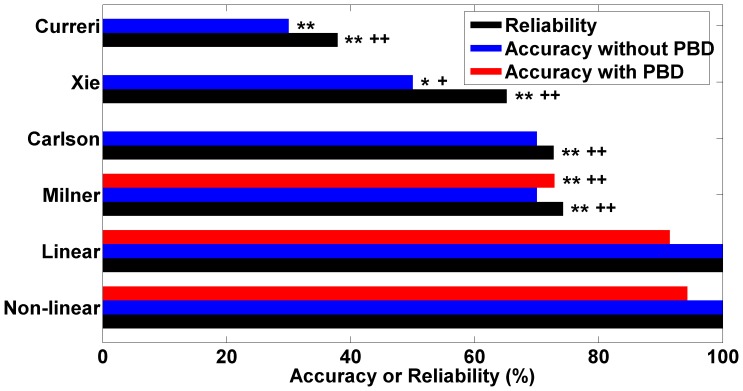
Comparison of accuracy and reliability of different formulas. “*” denotes comparison with the new non-linear estimation formula; “+” indicates comparison with the new linear estimation formula; “*” or “+” indicates P <0.05; “**” or “+ +” indicates P <0.01.

The comparisons of the estimates of energy consumption obtained from various formulas are shown in Table 6. When only TBSA was considered, the accuracies of the non-linear formula (4) and the linear formula (5) were both 100%; those of the commonly used Milner, Carlson, Xie, and Curreri formulas were 70%, 70%, 50%, and 30%, respectively (Figure 3). The chi-square test showed that only the Xie formula and the Curreri formula had significantly different accuracy from the newly built formulas (χ2  =  6.667, P  =  0.033; χ2  =  10.769, P  =  0.003 for nonlinear and linear formulas, respectively).

**Table 6:** Comparisons between the newly built formula and commonly used formulas with different combinations of PBD and TBSA.

TBSA (%)		REE(kcal·day^−1^)								Carlson	Xie	Curreri
		PBD 1d	PBD 2d	PBD 3d	PBD 7d	PBD 14d	PBD 21d	PBD 28d	Mean			
1–10	MEE	1758±126	1811±167	1889±133	1912±120	1874±66	1693±123	1617±93	1813±150	1546±164^b^	1710±90^b^	1596±120^b^
	Non-linear	1765±61^a^	1828±101^a^	1859±103^a^	1940±108^a^	1963±94^a^	1686±80^a^	1258±88^a*^	1796±208^b^			
	Linear	1782±61^a^	1826±100^a^	1842±101^a^	1902±104^a^	2046±100^a^	1676±63^a^	1517±63^a^	1821±161^b^			
	Milner	2017±170^a^	2062±212^a^	2055±211^a^	2031±209^a^	2063±175^a^	1928±170^a^	1903±186^a^	2020±189^b^			
11–20	MEE	1981±226	2028±225	2111±246	2146±212	2195±225	1995±243	1814±151	2045±239	1825±184^b^	1945±142^b^	1964±218^b^
	Non-linear	1965±138^a^	2006±142^a^	2034±145^a^	2148±157^a^	2164±167^a^	1960±164^a^	1553±150^a^	1989±232^b^			
	Linear	1986±141^a^	2002±142^a^	2009±143^a^	2083±147^a^	2195±154^a^	1876±142^a^	1719±132^a^	1993±194^b^			
	Milner	2343±210^a^	2337±209^a^	2346±214^a^	2303±206^a^	2255±202^a^	2222±203^a^	2174±200^a^	2286±204^b^			
21–30	MEE	2010±191	2055±198	2144±218	2241±214	2267±212	2025±70	1935±85	2097±203	2033±167^b^	2205±45^b^	2387± 66^b^
	Non-linear	2036±44^a^	2085±45^a^	2131±45^a^	2269±45^a^	2347±44^a^	2214±44^a^	1871±51^a^	2136±154^b^			
	Linear	2084±42^a^	2100±42^a^	2116±42^a^	2179±44^a^	2290±47^a^	2036±39^a^	1881±36^a^	2098±124^b^			
	Milner	2441±208^a*^	2434±208^a^	2427±207^a^	2401±205^a^	2354±201^a^	2307±196^a^	2261±192^a^	2375±198^b^			
31–40	MEE	2062±237	2119±218	2318±229	2394±219	2439±225	2389±228	2035±193	2279±258	2270±232^b^	2485±144^b^	2824±202^b*^
	Non-linear	2113±79^a^	2172±83^a^	2228±103^a^	2402±120^a^	2541±144^a^	2468±160^a^	2199±181^a^	2329±196^b^			
	Linear	2225±104^a^	2241±105^a^	2254±121^a^	2317±123^a^	2429±127^a^	2226±136^a^	2079±140^a^	2260±155^b^			
	Milner	2596±247^a*^	2589±246^a*^	2564±226^a^	2537±224^a^	2491±221^a^	2444±217^a^	2389±233^a^	2508±222^b^			
41–50	MEE	2098±289	2245±176	2401±211	2489±199	2676±216	2474±152	2195±172	2375±263	2442±253^b^	2710±157^b^	3197±236^b*^
	Non-linear	2119±189^a^	2208±184^a^	2269±189^a^	2472±208^a^	2661±227^a^	2639±229^a^	2406±216^a^	2403±272^b^			
	Linear	2300±215^a^	2335±199^a^	2351±200^a^	2415±205^a^	2526±215^a^	2366±205^a^	2209±193^a^	2359±210^b^			
	Milner	2647±291^a*^	2658±264^a^	2652±263^a^	2626±261^a^	2580±256^a^	2534±252^a^	2488±248^a^	2596±249^b^			
51–60	MEE	2050±171	2229±188	2356±180	2515±246	2693±244	2667±202	2487±229	2455±289	2745±217^b^	2983±134^b*^	3617±194^b*^
	Non-linear	2133±179^a^	2216±172^a^	2277±167^a^	2515±186^a^	2760±197^a^	2803±204^a^	2636±198^a^	2508±305^b^			
	Linear	2433±215^a^	2456±199^a^	2459±189^a^	2522±193^a^	2628±189^a^	2524±186^a^	2369±176^a^	2488±198^b^			
	Milner	2868±247^a*^	2893±240^a*^	2870±227^a*^	2843±225^a^	2779±213^a^	2732±210^a^	2685±206^a^	2802±223^b^			
61–70	MEE	1962±265	2074±227	2490±272	2694±256	2778±270	2836±275	2637±281	2535±391	2930±162^b^	3221±44^ *^	3995± 62^b*^
	Non-linear	2137±156^a^	2217±138^a^	2294±141^a^	2573±136^a^	2876±141^a^	2966±134^a^	2844±117^a^	2596±339^b^			
	Linear	2538±145^a*^	2561±127^a*^	2577±128^a^	2651±121^a^	2763±127^a^	2699±114^a^	2541±106^a^	2625±140^b^			
	Milner	2937±211^a*^	2934±182^a*^	2928±182^a^	2934±180^a^	2888±177^a^	2841±174^a^	2794±170^a^	2890±174^b^			
71–80	MEE	1707±109	1916±96	2139±78	2549±58	2692±68	2948±85	2872±83	2413±469	3030±265^b*^	3444±109^b*^	4365±144^b*^
	Non-linear	2063±68^a*^	2152±73^a^	2224±90^a^	2532±95^a^	2886±118^a^	3031±134^a^	2967±142^a^	2563±402^b^			
	Linear	2132±79^a*^	2184±81^a^	2223±97^a^	2444±91^a^	2808±105^a^	2933±116^a^	3065±121^a^	2553±378^b^			
	Milner	2983±246^a*^	2977±246^a*^	2946±294^a*^	2945±243^a^	2900±240^a^	2855±237^a^	2810±233^a^	2915±225^b*^			
81–90	MEE	1619±139	1976±167	2125±163	2580±273	2723±307	3017±343	3101±336	2332±576	3304±261^b*^	3720±112^b*^	4802±149^b*^
	Non-linear	1991±116^a*^	2056±94^a^	2148±100^a^	2508±200^a^	2917±230^a^	3118±245^a^	3110±244^a^	2450±490^b^			
	Linear	2127±123^a*^	2150±109^a^	2201±112^a^	2434±194^a^	2797±222^a^	2961±234^a^	3093±245^a^	2461±407^b^			
	Milner	3160±243^a*^	3121±249^a*^	3115±248^a*^	3065±296^a^	3020±292^a^	2975±287^a^	2930±283^a^	3071±254^b*^			
91–100	MEE	1493±114	1691±112	1987±122	2400±133	2616±141	2742±160	2851±171	2254±518	3401±251^b*^	3843±106^b*^	5006±155^b*^
	Non-linear	1822±117^a*^	1921±123^a^	2015±128^a^	2353±149^a^	2789±175^a^	3027±189^a^	3066±192^a^	2428±520^b^			
	Linear	2017±127^a*^	2067±130^a*^	2116±133^a^	2314±145^a^	2661±167^a^	2848±178^a^	2974±186^a^	2428±397^b^			
	Milner	3192±245^a*^	3185±244^a*^	3179±244^a*^	3154±242^a*^	3109±238^a^	3065±235^a^	3020±231^a^	3129±221^b*^			

**Note:** Data are presented as means ± SD. MEE, measured resting energy expenditure. The letter “a” is used to indicate the results of the comparisons between MEE and non-linear, linear or Milner estimating formula, the letter “b” is used to indicate the results of the comparisons between mean MEE and Carlson, Xie, or Curreri formulas. * indicates that the result does not lie in the range of ±20% for MEE.

When both TBSA and PBD were considered, we found that the accuracies of the new nonlinear formula (94.29%) and the linear formula (91.43%) were significantly higher than that of the Milner formula (72.86%) (χ2  =  11.706, P  =  0.001; χ2  =  8.230, P  =  0.004, respectively), whereas no significant difference in accuracy was noted between the non-linear formula and the linear formula (χ2  =  0.431, P  =  0.512) (Figure 3). Furthermore, for different TBSA or different combinations of TBSA and PBD, the estimation errors and the variation ranges of the new formulas were closer to zero than those of other formulas commonly used in the clinic, suggesting the new formulas are more accurate (See Table S1 and Table S2 for the details).

**Figure 4:**
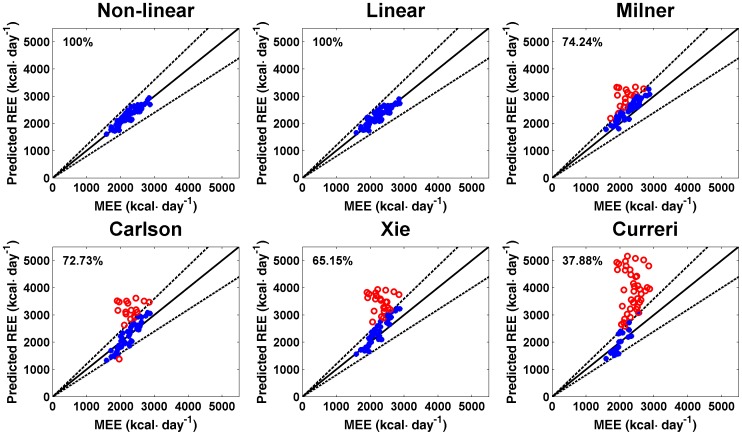
Reliabilities of different formulas. The solid line represents the ideal case of complete match between REE estimates and REE measurements (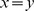), and the dashed lines represent 20% over or below the ideal match. Data points that fall between the two dashed lines are indicated by blue “*”; those outside are indicated by red “o”. Percentage represents the proportion of data points that fell between the two dashed lines. MEE, measured resting energy expenditure.

In addition, the overall reliabilities of the new estimation formulas were both 100% (Figure 4). Thus, the REE estimates for each of the 66 patients were located within the range of 20% above or below REE measurements. In contrast, as shown in Figure 4, the overall reliabilities of estimates obtained with the other formulas were significantly worse, with the Milner formula the most reliable (74.24%) and the Curreri formula the least (37.88%). The chi-square tests showed that the reliability of the Milner formula was also significantly lower than that of our new estimation formulas (χ2  =  19.513, P  =  0.000, Figure 3).

## Discussion

Severe burns lead to increased catabolism and higher energy consumption, and optimal patient care requires knowledge of the nutritional needs of the burn patients [21]–[26]. When underfed, patients may have complications that result in poor prognosis. However, over feeding increases metabolic burden and aggravates a patient's internal environment [27]–[32]. Although accurate knowledge of energy consumption in burn patients is necessary for development of a nutritional regimen, numerous factors result in observed energy metabolism after burn injury and most of the formulas currently available for energy consumption estimation suffer from limitations.

Our present study demonstrated that the extent of the burn (TBSA), post-burn days (PBD), and body surface area (BSA) were the main factors that affected REE in burn patients. TBSA and PBD are burn-related variables and are the core contributors to changes in energy metabolism after injury. Measurements of RRE in a group of 66 patients showed that the change in REE after burn was not a simple, straight line, but an S-shaped curve closely related to the TBSA, PBD, and BSA. Patients with different extents of burn showed significant differences in the REE changes with time. Starting REE values were higher in patients with moderate to severe burn (TBSA: 20–70%) than those with extremely severe burns, but rose more slowly, peaking 7–14 days after injury before declining slowly. In patients with extremely severe burn (TBSA> 70%), due to severe shock and metabolic inhibition, REE values were low at baseline, but surged after the shock stage (PBD 3–5), and generally peaked at PBD 21 before leveling off.

Given that most formulas currently available for energy consumption estimations are linear equations that fail to take into account the time factor, we built an estimation formula that contains PBD (post-burn days) as a core factor. By using a differential equation model and regression analysis, we generated a nonlinear equation, formula (4), for energy consumption estimation that incorporates TBSA and PBD. This formula offered significantly higher accuracy than formulas commonly used in clinical practice. As the nonlinear formula involves very complex calculations, we sought a simple formula that could be readily applied in clinical practice. Therefore, considering the impact of TBSA and PBD on REE, we converted the original non-linear estimation formula to four linear estimation formulas using piecewise linear fitting. The use of TBSA of 70% as the dividing point for the formula produced one formula for moderate and severe burn patients and another for patients with extremely severe burns. The use of PBD 14 as the dividing point for the formulas produced two additional equations. Through this linear fitting, we greatly simplified the estimation formulas while maintaining their accuracy. The four new formulas described are accurate and enable straightforward estimation of the energy needs of Chinese adults with burn injuries.

The energy consumption estimate based on the unit burn area (also known as the burn area-related coefficient) is the core determinant of the accuracy of energy estimation formulas. For example, according to the Xie formula, the energy consumption for every 1% TBSA is estimated to be 25 kcal. This formula is relatively accurate when used for energy estimates of patients with TBSA 30–70%, with an error of about 20%. However, for patients with TBSA ranging from 71% to 100%, the formula produces estimates about 40% higher than measured values[6], [11], [12]. The reason is that energy consumption does not have a simple linear relationship with burn area. When the burn area reaches a certain level, a further increase in burn area does not lead to proportional REE increases. When TBSA is greater than 70%, the estimated 25 kcal for each 1% TBSA is too high, and the error is significant. In the Curreri formula, the energy estimate for every 1% TBSA is 40 kcal, and estimates made using this formula are much higher than actual consumptions of severe burn patients. The Curreri formula was once the most commonly used energy estimation formula in Europe and the United States, but the utilization rate has declined from 60% to 4%, and it is no longer a commonly used formula[5], [7].

The extent to which the energy estimates based on unit burn area fits the change pattern of energy consumption in burn patients directly affects the accuracy of estimation formulas. We established two formulas that take into account the size of the burn area: one for patients with TBSA> 70% and the other for those with TBSA ≤ 70%. In the former estimation formula, the energy estimate per 1% TBSA is 7 kcal in the 14 days immediately after injury and 10 kcal at longer times post-burn. Based on this estimate REE will increase by 9.8–18 kcal (multiplied by BSA coefficient 1.4–1.8) for every 1% TBSA increase; this fits well with measured energy requirements for patients with moderate to severe burns. When TBSA is greater than 70%, REE changes as a result of an increase in burn area were insignificantly. Therefore, the coefficient of energy consumption per unit burn area was low at −0.4 and 2 in the formulas we built. Even when BSA is taken into account, energy consumption changes very little with each 1% increase in TBSA. A careful analysis of the change in pattern of REE in patients with extremely severe burn showed that REE peaked with TBSA at 80%, after which further increases in burn area resulted in a lower REE rather than a higher REE. For these patients, number of days post-burn is an important factor affecting REE. The time coefficients were 33 and 12 before and after PBD 14, respectively. Within the first 14 days after injury, REE increased by 46–60 kcal with each passing day, and after PBD14, REE increased by about 17–22 kcal until PBD 28. For patients with TBSA <70%, REE peaked at about PBD 14, before which the time coefficient was 10, indicating a daily increase of 14–18 kcal. REE showed a downward trend after PBD 14 for these patients, with a time coefficient of −14, which means that starting from PBD 15, REE was reduced by approximately 20–25 kcal with each passing day. In summary, this new simplified REE estimation formula, formula (5), fits well with the actual impact of the degree of burn and post-burn days on energy consumption in burn patients, providing accurate yet simple estimates of the actual energy needs of burn patients.

The Carlson formula provides a reasonable coefficient of burn area and its estimated values are also relatively close to the real values. However, the weakness of the formula is that it does not take into account post-burn days and, therefore, does not reflect the dynamic changes in energy consumption in burn patients[8]. The Milner formula is now recognized as the most accurate estimation formula currently in use clinically, it considers basal metabolic rate and the three core factors that affect energy consumption after burn: TBSA, PBD, and BSA[8], [21]. The weakness of the formula is that the coefficient of post-burn days is negative, suggesting gradually decreased energy estimates over time after burn, which is inconsistent with the marked rise in energy metabolism in the early stage after burn injury.

Based on clinical nutrition practice, this study adopted the range of 20% above or below actual REE measurements in burn patients as the numerical range for judging the accuracy and overall reliability of estimation formulas[6], [7], [17], [18]. A comparison of the nonlinear and linear equations we developed in this study with several other commonly used formulas showed that our new formulas had significantly higher overall reliability and accuracy than other formulas. Using the new formulas estimates for all patients fell within the range of 20% below or above the measured values. In contrast, the other formulas all generated estimates outside this range, and even the Milner formula, which is now recognized as the most accurate formula currently used, had a match rate of only 74.24%. The estimates from all the other formulas were higher than 20% of measured values, suggesting that these formulas significantly overestimate energy consumption by burn patients, especially those with severe burns. The formulas we established in this study provide accurate estimates of the energy consumptions of burn patients and will be of great value for the development of rational nutrition therapy programs to prevent overfeeding but ensure adequate nutritional supplementation.

Through long-term clinical observations, we have found that the response to burn injury is closely related to the degree of disease severity. Burn area is the most intuitive indicator of patient prognosis. Patients with TBSA> 70% show marked differences from patients with TBSA <70% in terms of pathophysiological reactions and have remarkably higher mortality rates[33], [34]. When it comes to energy consumption in burn patients, patients with TBSA <70% show higher starting REE, smaller increase, and a shorter time to peak than those with severe burns. In contrast, extremely severe burn patients have low starting REE, steep rises in energy requirements, and a long time to peak. Our energy estimation formula takes into account these characteristics of burn patients, and we use a burn area of 70% as the dividing point. Patients were further divided into two groups based on time since injury: up to 14 days and beyond 14 days. The four simplified energy estimation formulas accommodate the two key factors of the degree of burn and time after injury, making them relatively simple yet accurate.

The new energy estimation formulas we built for adult burn patients produce estimates very close to actual consumption with favorable accuracy and simplicity; however, the new formulas still suffer from some drawbacks. For example, the degree of burn is assessed by burn area alone. In fact, the depth of burn also exerts a very significant effect on metabolism. For patients with the same burn area, those with second-degree burns have different energy needs than those with third-degree burns. However, the determination of burn depth depends mainly on clinical experience. Due to the absence of objective and accurate tests, we did not incorporate burn depth as a variable.

Metabolic changes in burn patients are complicated and are regulated by numerous factors[1]–[3], [6]. Therefore, no formula can generate estimates completely consistent with actual energy consumptions in burn patients, and the formulas can only provide a rough range. Decision making on energy supply should be based on the specific conditions of the patients. In addition to considering predicted energy needs, physicians must also consider the metabolic ability of the patients. Patients' abilities to metabolize the energy supplied can be less than the energy consumption for quite some time after burn. With accurate formulas for prediction of energy needs, we can gain accurate insight into energy debt and cumulative energy imbalance of burn patients and provide reliable data for timely adjustment of nutrition regimens and prognosis prediction.

## Supporting Information

Table S1 Estimation error and its range in the newly built formula and commonly used formulas with different TBSA.
(DOC)

Table S2 Estimation error and its range in the newly built formula and commonly used formulas with different combinations of PBD and TBSA.
(DOC)

File S1 S3-RawData.xls. The original data in this study.
(XLS)
