# Supplementary material for: Establishment and Assessment of New Formulas for Energy Consumption Estimation in Adult Burn Patients
Source: PLoS One. 2014 Oct 16;9(10):e110409. doi: 10.1371/journal.pone.0110409 (PMC4199722; doi:10.1371/journal.pone.0110409)
Supplement: Table S1 — Estimation error and its range in the newly built formula and commonly used formulas with different TBSA. (DOC) [file pone.0110409.s001.doc]

Supplementary Table S1

Table S1. **Estimation error and its range in the newly built formula and commonly used formulas with different TBSA**.

| TBSA (%) | Error and its range | | | | | |
| --- | --- | --- | --- | --- | --- | --- |
|  | Non-linear | Linear | Milner | Carlson | Xie | Curreri |
| 1-10 | -17±166  (-212 to 147) | 8±144  (-225 to 155) | 208±192  (-152 to 473) | -281±164  (-577 to -28) | -116±112  (-302 to 1) | -231±115  (-386 to -75) |
| 11-20 | -56±141  (-198 to 82) | -52±128  (-215 to 85) | 241±162  (90 to 401) | -222±85  (-305 to -71) | -102±78  (-237 to -24) | -82±64  (-142 to 41) |
| 21-30 | 39±183  (-139 to 147) | 1±172  (-183 to 119) | 278±277  (-116 to 635) | -64±202  (-402 to 210) | 108±95  (-37 to 189) | 290±78  (184 to 382) |
| 31-40 | 50±134  (-139 to 176) | -19±147  (-190 to 125) | 229±204  (48 to 394) | -14±110  (-185 to 123) | 202±97  (79 to 329) | 541±76  (446 to 619) |
| 41-50 | 28±159  (-80 to 107) | -16±149  (-110 to 74) | 222±252  (-4 to 377) | 64±144  (-154 to 238) | 332±82  (228 to 460) | 819±94  (721 to 934) |
| 51-60 | 52±137  (-82 to 242) | 33±206  (-172 to 259) | 347±294  (86 to 590) | 281±152  (50 to 549) | 519±132  (311 to 726) | 1153±159  (882 to 1460) |
| 61-70 | 61±199  (-173 to 201) | 90±297  (-136 to 281) | 354±357  (200 to 471) | 375±124  (188 to 517) | 667±249  (356 to 1026) | 1441±269  (1094 to 1804) |
| 71-80 | 150±162  (6 to 301) | 140±202  (3 to 287) | 502±566  (166 to 733) | 614±269  (242 to 867) | 1028±139  (891 to 1221) | 1949±166  (1759 to 2165) |
| 81-90 | 118±167  (1 to 228) | 129±230  (-12 to 316) | 739±631  (382 to 1432) | 1050±423  (556 to 1619) | 1466±375  (901 to 1929) | 2548±376  (1953 to 3032) |
| 91-100 | 173±144  (97 to 265) | 174±206  (93 to 272) | 875±583  (632 to 1060) | 1147±189  (884 to 1329) | 1589±119  (1458 to 1717) | 2752±156  (2622 to 2943) |

**Note:** Data are presented as Mean ± SD. The values in brackets are the range of estimation error.
