# Supplementary material for: Establishment and Assessment of New Formulas for Energy Consumption Estimation in Adult Burn Patients
Source: PLoS One. 2014 Oct 16;9(10):e110409. doi: 10.1371/journal.pone.0110409 (PMC4199722; doi:10.1371/journal.pone.0110409)
Supplement: Table S2 — Estimation error and its range in the newly built formula and commonly used formulas with different combinations of PBD and TBSA. (DOC) [file pone.0110409.s002.doc]

Supplementary Table S2

Table S2. **Estimation error and its range in the newly built formula and commonly used formulas with different combinations of PBD and TBSA**.

| TBSA (%) | | Error and its range | | | | | | |
| --- | --- | --- | --- | --- | --- | --- | --- | --- |
|  |  | PBD 1d | PBD 2d | PBD 3d | PBD 7d | PBD 14d | PBD 21d | PBD 28d |
| 1-10 | Non-linear | 7±138  (-239 to 144) | 17±181  (-239 to 194) | -30±137  (-243 to 105) | 28±119  (-127 to 148) | 89±78  (-15 to 165) | -7±115  (-139 to 164) | -358±62  (-403 to -267) |
|  | Linear | 25±139  (-223 to 164) | 16±182  (-242 to 192) | -47±139  (-262 to 89) | -10±124  (-172 to 113) | 172±86  (54 to 259) | -18±119  (-160 to 152) | -99±63  (-163 to -21) |
|  | Milner | 260±200  (-106 to 545) | 251±247  (-145 to 611) | 166±207  (-184 to 459) | 118±191  (-172 to 395) | 189±150  (7 to 347) | 234±154  (37 to 446) | 286±168  (138 to 508) |
| 11-20 | Non-linear | -16±128  (-238 to 184) | -21±121  (-191 to 186) | -77±127  (-274 to 60) | 2±123  (-156 to 135) | -31±98  (-213 to 100) | -35±111  (-201 to 119) | -261±146  (-480 to -48) |
|  | Linear | 5±125  (-209 to 204) | -26±121  (-195 to 180) | -102±129  (-302 to 34) | -64±127  (-233 to 82) | 0±110  (-195 to 153) | -119±134  (-332 to 48) | -94±138  (-308 to 112) |
|  | Milner | 363±118  (250 to 539) | 309±113  (210 to 463) | 234±127  (85 to 419) | 156±83  (43 to 281) | 60±100  (-80 to 232) | 227±136  (72 to 403) | 361±213  (-42 to 520) |
| 21-30 | Non-linear | 26±206  (-267 to 225) | 30±208  (-291 to 212) | -13±221  (-319 to 160) | 28±199  (-251 to 235) | 80±188  (-162 to 295) | 189±86  (64 to 282) | -64±108  (-235 to 33) |
|  | Linear | 74±197  (-210 to 261) | 45±202  (-270 to 221) | -28±216  (-329 to 136) | -62±200  (-341 to 148) | 24±198  (-232 to 236) | 11±84  (-105 to 115) | -55±106  (-219 to 72) |
|  | Milner | 430±302  (-68 to 861) | 379±297  (-148 to 735) | 283±301  (-229 to 688) | 160±273  (-328 to 461) | 87±262  (-370 to 369) | 283±224  (13 to 631) | 325±246  (8 to 696) |
| 31-40 | Non-linear | 51±165  (-176 to 214) | 53±140  (-151 to 155) | -91±143  (-369 to 55) | 8±120  (-214 to 178) | 102±100  (-84 to 219) | 79±95  (-54 to 204) | 164±66  (75 to 256) |
|  | Linear | 163±140  (-33 to 296) | 122±120  (-56 to 198) | -65±128  (-318 to 63) | -77±116  (-291 to 88) | -9±114  (-215 to 123) | -164±110  (-324 to -19) | 44±79  (-89 to 129) |
|  | Milner | 534±122  (383 to 653) | 470±100  (340 to 563) | 245±133  (88 to 416) | 143±131  (-23 to 312) | 52±135  (-101 to 264) | 55±103  (-57 to 219) | 354±118  (222 to 500) |
| 41-50 | Non-linear | 22±139  (-190 to 195) | -37±123  (-159 to 174) | -132±100  (-325 to -48) | -17±97  (-188 to 74) | -15±69  (-112 to 69) | 166±141  (-46 to 344) | 211±157  (40 to 468) |
|  | Linear | 203±114  (19 to 332) | 90±118  (-62 to 279) | -51±89  (-217 to 12) | -75±87  (-230 to 5) | -150±65  (-248 to -84) | -108±123  (-284 to 67) | 15±135  (-116 to 254) |
|  | Milner | 549±127  (365 to 674) | 413±194  (69 to 601) | 251±156  (30 to 409) | 136±140  (-60 to 275) | -97±131  (-269 to 50) | 60±193  (-273 to 288) | 293±193  (-5 to 584) |
| 51-60 | Non-linear | 83±126  (-116 to 245) | -13±96  (-192 to 124) | -79±90  (-195 to 38) | 0±142  (-172 to 257) | 67±147  (-175 to 334) | 136±97  (2 to 350) | 149±119  (13 to 345) |
|  | Linear | 383±135  (231 to 583) | 227±94  (93 to 398) | 103±92  (3 to 253) | 7±143  (-135 to 280) | -66±148  (-308 to 196) | -143±95  (-271 to 54) | -118±119  (-277 to 51) |
|  | Milner | 818±146  (611 to 1047) | 664±126  (506 to 838) | 514±129  (336 to 669) | 328±144  (195 to 600) | 86±159  (-164 to 348) | 65±120  (-75 to 258) | 198±143  (1 to 475) |
| 61-70 | Non-linear | 175±120  (38 to 291) | 142±115  (-41 to 264) | -196±144  (-411 to -45) | -121±140  (-384 to -2) | 98±146  (-166 to 253) | 130±157  (-132 to 317) | 206±190  (-119 to 398) |
|  | Linear | 576±137  (420 to 704) | 486±134  (274 to 616) | 87±161  (-158 to 248) | -43±158  (-335 to 98) | -15±160  (-298 to 164) | -137±177  (-426 to 87) | -96±197  (-429 to 118) |
|  | Milner | 975±71  (895 to 1050) | 860±91  (725 to 966) | 438±107  (268 to 533) | 241±127  (-6 to 344) | 110±133  (-137 to 216) | 5±132  (-229 to 139) | 157±148  (-115 to 271) |
| 71-80 | Non-linear | 356±154  (177 to 551) | 236±139  (80 to 411) | 85±143  (-56 to 229) | -17±124  (-178 to 113) | 194±109  (69 to 332) | 83±103  (-22 to 222) | 95±114  (-25 to 247) |
|  | Linear | 424±165  (229 to 631) | 268±148  (100 to 453) | 84±150  (-65 to 236) | -105±121  (-261 to 23) | 116±96  (11 to 240) | -15±87  (-95 to 106) | 193±92  (101 to 319) |
|  | Milner | 1276±316  (834 to 1567) | 1061±297  (650 to 1328) | 807±331  (430 to 1051) | 396±259  (15 to 599) | 208±213  (-96 to 397) | -93±183  (-350 to 80) | -62±183  (-319 to 111) |
| 81-90 | Non-linear | 372±45  (282 to 419) | 80±86  (-51 to 187) | 24±79  (-71 to 167) | -72±119  (-193 to 85) | 193±96  (96 to 325) | 101±116  (7 to 266) | 9±104  (-69 to 154) |
|  | Linear | 508±43  (444 to 584) | 174±82  (61 to 266) | 77±76  (-4 to 209) | -146±119  (-262 to 16) | 74±105  (-38 to 214) | -56±127  (-172 to 120) | -8±107  (-113 to 137) |
|  | Milner | 1541±160  (1254 to 1760) | 1146±176  (895 to 1367) | 990±184  (678 to 1168) | 485±193  (475 to 534) | 296±163  (122 to 515) | -42±185  (-200 to 226) | -171±170  (-310 to 78) |
| 91-100 | Non-linear | 329±56  (273 to 407) | 230±41  (196 to 288) | 28±47  (-20 to 92) | -47±77  (-147 to 39) | 173±79  (85 to 277) | 285±106  (164 to 422) | 215±86  (124 to 327) |
|  | Linear | 524±78  (442 to 629) | 376±59  (319 to 458) | 129±63  (62 to 213) | -86±84  (-191 to 10) | 45±74  (-35 to 144) | 106±97  (0 to 234) | 123±70  (51 to 216) |
|  | Milner | 1699±187  (1428 to 1858) | 1495±198  (1251 to 1682) | 1192±185  (933 to 1372) | 754±159  (530 to 907) | 493±184  (249 to 692) | 323±170  (106 to 511) | 169±179  (-38 to 397) |

**Note:** Data are presented as Mean ± SD. The values in brackets are the range of estimation error.
